# Prediction of carcass composition through measurements *in vivo* and measurements of the carcass of growing Santa Inês sheep

**DOI:** 10.1371/journal.pone.0247950

**Published:** 2021-03-05

**Authors:** Mariléa Batista Gomes, Maria Luciana Menezes Wanderley Neves, Lígia Maria Gomes Barreto, Marcelo de Andrade Ferreira, João Paulo Ismério dos Santos Monnerat, Guilherme Morais Carone, Jasiel Santos de Morais, Antonia Sherlânea Chaves Véras

**Affiliations:** 1 Department of Animal Science, Federal Rural University of Pernambuco, UFRPE, Recife, Pernambuco, Brazil; 2 Department of Animal Science, Federal University of Sergipe, Nossa Senhora da Glória, Sergipe, Brazil; Tokat Gaziosmanpasa Universitesi, TURKEY

## Abstract

In *vivo* and carcass measurements were evaluated to predict carcass physical and chemical composition and to list the measurements that best fit the prediction of the composition of growing Santa Inês sheep carcasses. Thirty-three animals were used to measure the loin eye area by ultrasound *in vivo* (LEAu) and in the carcass. We used 39 animals for biometric measurement *in vivo* and 42 sheep for morphometric measurement in the carcass. For the physical and chemical compositions of carcasses, dissection of the half left carcass was carried out in 42 animals. The data were submitted to Pearson’s correlation analysis and t test. Simple and multiple linear regressions were performed using a stepwise procedure. All correlations between *in vivo* measurements and the physical and chemical compositions of carcasses (in kg) were significant, except for LEAu. Biometric measurements and hot (HCW) and cold (CCW) carcass weights were considered as predictors of the carcasses’ physical and chemical compositions. Slaughter body weight (SBW) was the variable that most influenced the equations in the assessment of *in vivo* measurements and HCW and CCW most influenced the equations for measurements on carcasses. Biometric measurements of Santa Inês sheep can be used together with the SBW to estimate the physical and chemical compositions of carcasses, with emphasis on body compactness index, breast width, wither height, and croup height. The morphometric measurements can be used together with carcass weight to estimate the physical and chemical compositions of carcasses, with emphasis on croup width, carcass compactness index, croup perimeter, external and internal carcass lengths, chest width, and leg length and perimeter. The HCW can be used to predict the physical and chemical composition of carcasses without affecting the accuracy of the prediction model.

## Introduction

The management of a breeding system associated with factors inherent to the animals and the environment directly affect the production rates [1]. In this context, native breeds, in addition to their genetic and cultural value, are a source of income, employment, and food security for low-income farmers [2], which is the case in northeastern Brazil [3].

The adoption of indirect methods to predict carcass components offers the possibility to gain a subjective knowledge of carcass composition. It allows to follow the growth and development of an animal of interest to the meat industry within the scope of precision management of herds [4]. Thus, such tools can enable the producer to gain control and interference regarding the composition of the final product, seeking to meet the demands of the consumer market [5], and to determine the real nutritional deficiency of growing animals and obtain gains in weight. In this sense, the development of fast and reliable methods to predict the physical and chemical composition of the carcass in ruminants can help the producer to obtain data in vivo to predict carcass composition, with view to making decisions related to animal growth to meet consumer market demands, as well as providing knowledge of the composition of the carcass without the need to dissect it.

Additionally, *in vivo* assessments are non-invasive, less laborious, quick, and low-cost techniques compared to carcass measurements. According to Diaz et al. [6], body weight can easily be measured in the field and is a low-cost approach compared to the use of ultrasound. However, although weight measurement is of fundamental importance for meat production systems, it should not be the only measurement considered, since it does not represent the standardization required by the consumer in relation to the quality of cuts.

Real-time ultrasound images can predic tissue composition and widely used because of their high correlation with carcass muscle and fat composition, which is easily measured in the muscle *Longissimus lumborum* between the 12th and 13th thoracic vertebrae [7]. However, their use may be limited by breed specificities, small tissue quantities, and the use of measurements not routinely performed.

Thus, *in vivo* assessment techniques must be highly accurate. The predictive models generated from real-time measurements must also be highly accurate, so that the components of the carcass can be estimated with accuracy.

Thus, we hypothesize that the combination of measurements performed on different parts of the body of a live animal and/or on the carcass could generate accurate models for predicting the composition of growing Santa Inês sheep carcasses. Therefore, we evaluated *in vivo* and carcass measurements as factors to predict carcass physical and chemical compositions and list the method and/or measurements that best fit the prediction of the composition of growing Santa Inês sheep carcasses.

## Material and methods

### Experimental site, animals, and diets

The experimental procedures performed with the animals were approved by the Ethics Committee on Use of Animals of the Federal Rural University of Pernambuco (UFRPE), in the city of Recife, PE, Brazil, approval number 120/2017.

In total, was used 42 male Santa Inês sheep, non-castrated, aged between 6 and 7 months, with an initial mean body weight (BW) of 19.48 ± 1.86 kg, confined in individual pens provided with a feeder and a drinking fountain for 77 days. The diet followed a roughage:concentrate ratio of 50:50 to meet the nutritional requirements of sheep, aiming at an average daily gain of 200 g [8]. The roughage was Tifton hay and concentrate ground corn, soybean meal, candy industry residues associated with corn gluten meal, and mineral supplement.

### Ultrasound and biometric measurements

Measurements of animals, as well as of carcasses, were conducted after 77 experimental days. Ultrasound measurements were performed to obtain the loin eye area (LEAu), on the muscle *Longissimus lumborum*, in 33 animals in the region between the 12th and the 13th ribs using the Áquila Vet equipment from Esaote, Europe, at a wave frequency of 3.5 MHz. The sheep were manually immobilized, and the site was prepared (trichotomy) and cleaned. Vegetable oil was placed for acoustic contact and perfect transducer coupling, arranged perpendicularly to the length of the muscle *Longissimus lumborum* [4, 9]. A digital image of each animal was stored; it was digitized and subsequently measured using the software IMAGEJ^®^. All ultrasound records of animals were performed by the same operator and using the same equipment.

Biometric measurements were measured in 39 animals using a graduated plastic measuring tape and a compass. The animals were placed in an upright position with perpendicular anterior and posterior limbs over a flat surface. The following parameters were measured: body length (BL) [3], wither height (WH), croup height (CH), thoracic perimeter (TP_l_), croup width (CW_l_), and chest width (ChW). The body compactness index (BCI) was estimated by the relation between two measurements: slaughter body weight (SBW) and animal BL: BCI = SBW/BL (kg/cm) [10].

### Slaughter of experimental animals and morphometric measurements

After 77 days and after fasting of solids for 16 hours, the 42 animals were slaughtered [11] and weighed to obtain the SBW.

Stunning was performed by electronarcosis with 220 V of electric current and 1.5 Amps for 3 seconds. Then, bleeding was performed through a jugular and carotid section, and the animals were skinned and eviscerated.

The head, paws, penis, and testicles were removed, and the carcasses were weighed to obtain the hot carcass weight (HCW). Subsequently, they were stored in a cold chamber at 4°C for 24 hours to obtain the cold carcass weight (CCW).

Morphometric measurements were performed on the cooled carcass of the 42 animals. The carcasses were divided longitudinally on the dorsal midline to obtain and then weigh the half carcasses. The internal measurements of carcasses were determined in the cooled left half carcass suspended by the calcaneus tendon. Measurements of external carcass length (ECL), croup perimeter (CP), internal carcass length (ICL), and leg length (LL) were taken using a flexible graduated plastic measuring tape [10].

Additionally, using a compass and a measuring tape, the following measurements were taken: thoracic depth (TD), croup width (CWc), and thoracic width (TW). Carcass compactness index (CCI), which was obtained through the relation between CCW and ICL, and leg compactness index (LCI), which consists of the relation between CWc and LL, were determined. In addition, leg circumference (LC) and thoracic perimeter (TPc) [9] were measured.

In the carcass, the loin eye area (LEAc) was obtained by the cross sectioning of the muscle *Longissimus lumborum*. It consists of the measurements between the 12th and 13th thoracic vertebrae, recording and archiving the shape from the outline of the muscle’s transverse surface with transparent plastic film, using a pen. Subsequently, the sheets were digitized for area evaluation (cm^2^), using the software IMAGEJ^®^ [12, 13].

It is worth mentioning that all ultrasound, biometric, and morphometric records were performed by the same operator using the same equipment for uniformity purposes.

### Evaluations of the physical and chemical composition of carcasses

The physical evaluation of carcasses was conducted in the meat laboratory of the Animal Science Department of UFRPE/Recife, PE, Brazil, in a refrigerated environment. The half left carcasses of the 42 animals were individually placed in plastic bags, vacuum-packed, and frozen.

Subsequently, they were removed from the freezer and thawed in a refrigerator at 4°C until total thawing. Then, the muscle, bone, and adipose tissues of all meat cuts of the half left carcass [3] were dissected and quantified. Other tissues (blood and lymph vessels, lymph nodes, tendons, nerves, and connective tissues associated with muscle) were also quantified [10].

The tissues were weighed on a semi-analytical balance, and their weights were summed to obtain the reconstituted half carcass weight (RHC) and to later estimate their proportions in carcasses (%tissue = dissected tissue weight / RHC). To estimate the tissue weight in the entire carcass, the percentage of each tissue was multiplied by the CCW (in kg).

For the chemical composition, all tissues were ground and homogenized. For this, 150 g of sample was placed in a glass jar with a known mass and pre-dried in an oven at 65°C to obtain the fat dry matter (FDM). Subsequently, these samples were submerged in petroleum ether and stirred. Successive washes were conducted, resulting in pre-degreased dry matter (DDM). The fat removed in pre-degreasing was calculated as the difference between the FDM and DDM.

Then, the pre-degreased samples were ground in a ball mill to later determine dry matter (DM), according to the method 950.46 [14] and ash by the method 920.153 [14]. Residual fat (F) was extracted according to the method 960.39 [14], and total nitrogen (N) was assessed via the Kjeldahl method, method 928.080 [14]. A conversion factor of 6.25 was used to estimate the protein. The fat extracted in the pre-degreasing was added to the fat from the analysis of residual fat to obtain the total fat content in the samples (%). By knowing the contents of protein, fat, water, and ash, and the sample weight submitted to pre-degreasing, the respective contents in natural matter were determined. Then, these levels were multiplied by the CCW to obtain the respective quantities in carcasses.

### Data analysis

Statistical analyses were performed using the SAS statistical package [15]. Descriptive analysis was performed using the MEANS procedure. Correlations were obtained by Pearson’s correlation analysis (PROC CORR) and t test, considering a significance of P < 0.05. The criteria for the classification of correlation coefficients were r ≥ 70%, meaning a strong association, and 30% < r ≤ 70%, representing a moderate correlation [16].

Correlations were made between the physical and chemical constituents of carcasses. Subsequently, data on physical and chemical compositions were correlated with biometric, morphometric, as well as LEAu and LEAc measurements.

Simple linear regressions were used to estimate the functional relationships between variables. Multiple regression was applied to determine which variables can predict the chemical and physical composition of Santa Inês sheep carcasses. Biometric measurements with SBW and morphometric measurements with HCW or CCW were considered as possible independent variables in the study of the models.

The stepwise procedure was used to select the variables for prediction equations. The criteria used to choose the equations were model significance (P < 0.05), coefficient of determination (R^2^), and the residual standard deviation.

## Results

The descriptive statistics of measurements taken in live animals (Table 1) and after slaughter (Table 2) resulted in means, data amplitude, and variability represented by the mean standard error.

**Table 1:** Descriptive analysis of the means, standard error of mean (MSE), minimum (Min), and maximum (Max) of biometric measurements in Santa Inês sheep.

Variables	n	Means	MSE	Min	Max
Slaughter body weight, kg	39	34.358	0.831	18.290	40.500
Croup height, cm	39	67.385	0.547	57.000	73.000
Wither height, cm	39	68.000	0.396	61.000	72.000
Body length, cm	39	70.667	0.771	55.000	79.000
Chest width, cm	39	22.074	0.304	16.000	25.500
Croup width, cm	39	21.282	0.406	14.000	28.000
Thoracic perimeter, cm	39	74.244	0.536	64.000	81.000
Body compactness index, kg / cm	39	0.472	0.008	0.340	0.546
LEAu, cm^2^	33	12.949	0.331	9.203	17.084

n: number of animals, LEAu: Loin eye area obtained by ultrasound in the *Longissimus lumborum*.

**Table 2:** Descriptive analysis of the means, standard errors of mean (MSE), minimum (Min), and maximum (Max) of the measurements and physical and chemical compositions in the carcasses of Santa Inês sheep.

Variables	n	Means	MSE	Min	Max
**Weights**					
Hot carcass weight, kg	42	16.009	0.520	6.140	19.380
Cold carcass weight, kg	42	15.195	0.503	5.540	18.460
**Carcass measurements**					
External carcass length, cm	42	57.071	0.582	45.000	63.000
Internal carcass length, cm	42	56.190	1.076	45.000	68.000
Croup width, cm	42	24.048	0.267	19.500	27.000
Thoracic width, cm	42	22.655	0.351	18.000	26.500
Croup perimeter, cm	42	64.012	0.662	50.500	71.000
Leg length, cm	42	41.238	0.365	35.000	45.500
Thoracic depth,cm	42	25.119	0.228	21.000	28.000
Leg circumference, cm	42	40.095	0.613	24.000	46.000
Thoracic perimeter, cm	42	69.060	0.706	56.500	75.000
Carcass compactness index, kg / cm	42	0.273	0.010	0.111	0.360
Leg compactness index, kg / cm	42	0.583	0.005	0.500	0.658
LEAc, cm^2^	33	13.563	0.380	10.130	19.160
**Corrected physical composition in cold carcass**					
Muscle tissue, kg	42	9.094	0.297	3.137	11.290
Adipose tissue, kg	42	2.293	0.109	0.349	3.091
Bone tissue, kg	42	3.175	0.094	1.692	3.979
Other tissue, kg	42	0.632	0.028	0.201	0.995
Muscle tissue, %	42	59.924	0.315	56.411	65.057
Adipose tissue, %	42	14.666	0.444	6.296	18.511
Bone tissue, %	42	21.239	0.399	18.115	33.453
Other tissues, %	42	4.171	0.124	2.369	5.765
**Corrected chemical composition in cold carcass**					
Fat, kg	42	2.406	0.104	0.379	3.134
Protein, kg	42	3.105	0.108	1.092	4.259
Ash, kg	42	0.586	0.017	0.289	0.818
Water, kg	42	9.097	0.288	3.781	11.280
Fat, %	42	15.489	0.356	6.837	20.478
Protein, %	42	20.430	0.211	16.926	23.483
Ash, %	42	3.931	0.082	3.128	5.475
Water, %	42	60.150	0.372	55.872	68.241

n: number of animals, LEAc: Loin eye area obtained in the carcass after cross sectioning of the *Longissimus lumborum*.

All tissue components correlated positively with the chemical components of carcasses (Table 3). The amount of ash strongly correlated with bone tissue and, in a moderate way, with muscle and adipose tissues. The other chemical components correlated strongly with all tissues.

**Table 3:** Correlation coefficient between the quantities of tissue and chemical components of carcasses of Santa Inês sheep.

Chemical composition, kg	Muscle tissue, kg	Adipose tissue, kg	Bone tissue, kg
Fat	0.8343***	0.8953***	0.8009***
Protein	0.8952***	0.7936***	0.8535***
Ash	0.6972***	0.4863**	0.7897***
Water	0.9761***	0.8060***	0.8923***

**P < 0.01;

***P < 0.001.

Table 4 shows that all correlations between *in vivo* measurements and the physical and chemical compositions of carcasses (in kg) were significant, except for LEAu, which correlated significantly with muscle tissue (0.6662) as well as protein (0.4145) and water (0.5906) contents in carcasses. Correlations of adipose tissue were strong only with SBW, TP_l_, and BCI. The BCI was the parameter which was most strongly correlated with the physical and chemical compositions of animal carcasses, except for the correlation with other tissues and ash. Ash was strongly related to SBW and CH.

**Table 4:** Correlation coefficients between *in vivo* measurements and corrected physical and chemical compositions in cold carcasses of Santa Inês sheep.

*in vivo* measurements	Physical composition, kg				Chemical composition, kg			
	Muscle	Adipose	Bone	Others Tissues	Fat	Protein	Ash	Water
Slaughter body weight, kg	0.9459***	0.8408***	0.9022***	0.6754***	0.9027***	0.8912***	0.7011***	0.9585***
Croup height, cm	0.7834***	0.6650***	0.8083***	0.4992**	0.6943***	0.7617***	0.7016***	0.7999***
Wither height, cm	0.6125***	0.4952**	0.7012***	0.4731**	0.5584**	0.6161***	0.6366***	0.6330***
Body length, cm	0.7786***	0.6216***	0.7961***	0.5235**	0.6517**	0.7707***	0.6647***	0.7906***
Chest width, cm	0.7806***	0.5427***	0.7624***	0.4871**	0.6282***	0.6748***	0.6185***	0.7932***
Croup width, cm	0.6307***	0.4429**	0.5948***	0.5247***	0.5213***	0.4963**	0.4458**	0.6666***
Thoracic perimeter, cm	0.7984***	0.7731***	0.7774***	0.5298**	0.8323***	0.7728***	0.5232**	0.8064***
Body compactness index, kg / cm	0.8384***	0.7931***	0.8090***	0.6112***	0.8655***	0.7720***	0.5966***	0.8583***
LEAu, cm^2^	0.6662***	0.1985^ns^	0.2457^ns^	0.1573^ns^	0.1977^ns^	0.4145**	0.2770^ns^	0.5906***

^ns^P > 0.05;

**P < 0.01;

***P < 0.001.

LEAu: Loin eye area obtained by ultrasound in the *Longissimus lumborum*.

The correlations between *in vivo* measurements and compositions (expressed in %) were mostly not significant. There were moderate and positive correlations between the SBW and proportions of adipose tissue and fat in carcasses, in addition to a moderate negative association between SBW and proportions of muscle tissue and ash. The proportion of ash was the only chemical component that correlated in a moderate and negative way with most measurements, except for WH and LEAu, which were not significant (Table 5).

**Table 5:** Correlation coefficients between *in vivo* measurements and corrected proportions of physical and chemical compositions in cold carcasses of Santa Inês sheep.

*in vivo* measurements	Physical composition, %				Chemical composition, %			
	Muscle	Adipose	Bone	Others tissues	Fat	Protein	Ash	Water
Slaughter body weight, kg	- 0.3784*	0.4149**	- 0.1108^ns^	- 0.5550^ns^	0.4229**	- 0.0692^ns^	- 0.6302***	- 0.1223^ns^
Croup height, cm	- 0.3219*	0.2940*	0.0458^ns^	- 0.0384^ns^	0.2455^ns^	- 0.0400^ns^	- 0.42093**	- 0.0553^ns^
Wither height, cm	- 0.2964^ns^	0.1507^ns^	0.1845^ns^	0.0401^ns^	0.1892^ns^	- 0.0507^ns^	- 0.24825^ns^	- 0.0485^ns^
Body length, cm	- 0.2879^ns^	0.2364^ns^	0.0630^ns^	- 0.0012^ns^	0.1735^ns^	0.0033^ns^	- 0.44554**	- 0.0219^ns^
Chest width, cm	- 0.1173^ns^	0.0685^ns^	0.0689^ns^	- 0.0009^ns^	0.1445^ns^	- 0.1700^ns^	- 0.4126**	0.1329^ns^
Croup width, cm	- 0.1160^ns^	0.0416^ns^	0.0107^ns^	0.1696^ns^	0.1339^ns^	- 0.2668^ns^	- 0.3912*	0.2138^ns^
Thoracic perimeter, cm	- 0.3930*	0.4281**	- 0.0775^ns^	- 0.0007^ns^	0.4722**	- 0.0191^ns^	- 0.62158***	- 0.2070^ns^
Body compactness index, kg / cm	- 0.3991^ns^	0.4357**	- 0.1173^ns^	0.0641^ns^	0.4914**	- 0.1115**	- 0.5956***	- 0.1564^ns^
LEAu, cm^2^	0.3387^ns^	- 0.2038^ns^	- 0.2390^ns^	- 0.0522^ns^	- 0.2900^ns^	0.0029^ns^	- 0.0892^ns^	0.2654^ns^

^ns^P > 0.05;

*P < 0.1;

**P < 0.01;

***P < 0.001.

LEAu: Loin eye area obtained by ultrasound in the *Longissimus lumborum*.

Since the best associations of *in vivo* measurements were obtained with the compositions expressed in kg, the models were generated only with the compositions expressed in kg, using the SBW and biometric measurements as predictor variables (Table 6). Table 6 also shows the effect of including independent variables on the determination coefficient (R^2^) and the residual standard deviation.

**Table 6:** Prediction equations for the calculation of physical and chemical components *in vivo* from measurements in Santa Inês sheep.

Dependent variable Y	Steps	Independent variable X	P	R^2^	RSD	Equations
Muscle, kg	1	SBW	< .0001	0.8947	0.20002	Y = 0.95986 + 0.24782 SBW
	2	BCI	< .0001	0.9043	0.18671	Y = 1.94868 + 0.31088S BW– 6.68191 BCI
	3	ChW	< .0001	0.9146	0.17152	Y = 0.686664 + 0.28858SBW+ 0.11405 ChW– 7.7185 BCI
Adipose, kg	1	SBW	< .0001	0.707	0.08208	Y = − 0.47775 + 0.08461 SBW
	2	ChW	< .0001	0.7326	0.07699	Y = 0.37319 + 0.10390 SBW– 0.06856 ChW
Bone, kg	1	SBW	< .0001	0.814	0.05069	Y = 0.19492 + 0.08954 SBW
	2	WH	< .0001	0.8482	0.04253	Y = − 2.63808 + 0.07521 SBW + 0.04891 WH
Fat, kg	1	SBW	< .0001	0.8149	0.04301	Y = − 0.30112 + 0.08273S BW
Protein, kg	1	SBW	< .0001	0.7942	0.05829	Y = 0.13959 + 0.09017 SBW
	2	BCI	< .0001	0.8138	0.0542	Y = 0.68369 + 0.12487 SBW– 3.67676 BCI
Ash, kg	1	CH	< .0001	0.4923	0.00353	Y = − 0.53228 + 0.01689 CH
	2	ChW	< .0001	0.5488	0.00322	Y = − 0.51977 + 0.01244 CH + 0.01303 ChW
Water, kg	1	SBW	< .0001	0.9188	0.16388	Y = 0.54939 + 0.25894 SBW
	2	ChW	< .0001	0.9269	0.15172	Y = − 0.73160 + 0.22991 SBW + 0.10322 ChW
	3	BCI	< .0001	0.9352	0.13834	Y = 0.02779 + 0.28639 SBW + 0.11902 ChW– 6.45694 BCI

Steps: stepwise system steps; P: probability; R^2^: coefficient of determination; RSD: residual standard deviation; SBW: slaughter body weight; BCI: body compactness index; ChW: chest width; WH: wither height; CH: croup height.

"Other tissues" was the physical component that showed low correlations (kg) or non-significant correlations (%) with *in vivo* measurements (Tables 4 and 5). Asit is a physical component of little interest and difficult to separate from the others, no models were generated to estimate it.

For muscle tissue, the data fitted better the model by using multiple regression through the evaluation of coefficients of determination (R^2^), considering the SBW, BCI, and ChW (Table 6). For adipose tissue, SBW and ChW were added to the stepwise procedure, while for bone tissue, SBW and WH were added.

Regarding the chemical composition, the variable SBW was the most expressive one in terms of fat composition. It is also part of the equations related to protein and water. In addition, the BCI was included in the protein model, while ChW and BCI participated in the water prediction equation. The CH and ChW were included in the models referring to ash (Table 6).

Because LEAu correlated with the amounts of muscle tissue, protein, and water in carcasses (Table 4), it was inserted as a probable predictor only for these components. This measurement was used as a predictor in multivariate models for estimating muscle tissue (Mt, kg = 4.71053 + 0.22299 SBW + 0.1884 LEAu—0.0702 TP_l_, R^2^ = 0.8256) and carcass water (Cw, kg = 3.2059 + 0.25424 SBW + 0.1376 LEAu -0.05733 TP_l_, R^2^ = 0.8394), contributing to an increase of 22.94 and 10.52%, respectively, in model accuracy.

The coefficients of correlation between measurements on carcasses and physical and chemical compositions (expressed in kg) demonstrate that ICL, LCI, and LEAc did not significantly associate with physical and chemical compositions of carcasses, except for LEAc with muscle tissue and water, which presented a moderate correlation. Regarding ICL, the parameters bone tissue and ash composition were moderately correlated. The other measurements on carcasses showed positive strong to moderate correlations with the chemical and physical components of carcasses (Table 7).

**Table 7:** Correlation coefficients between carcass measurements and corrected physical and chemical compositions on the cold carcass of Santa Inês sheep.

Carcass measurements	Physical composition, kg				Chemical composition, kg			
	Muscle	Adipose	Bone	Other tissues	Fat	Protein	Ash	Water
**Weights**								
Hot carcass weight, kg	0.9879***	0.9226***	0.9280***	0.7353***	0.9485***	0.9559***	0.8517***	0.9884***
Cold carcass weight, kg	0.9883***	0.9282***	0.9319***	0.7469***	0.9516***	0.9594***	0.8575***	0.9906***
**Carcass measurements**								
External carcass length, cm	0.8594***	0.7826***	0.8223***	0.6511***	0.8213***	0.8512***	0.8136***	0.8481***
Internal carcass length, cm	0.2612^ns^	0.0765^ns^	0.3273*	0.2004^ns^	0.1701^ns^	0.2411^ns^	0.3127*	0.2537^ns^
Croup width, cm	0.8094***	0.6862***	0.7145***	0.6241***	0.6918***	0.7537***	0.6492***	0.8153***
Thoracic width, cm	0.8174***	0.7216***	0.8054***	0.6976***	0.7323***	0.7511***	0.7048***	0.8568***
Croup perimeter, cm	0.8760***	0.7693***	0.8410***	0.7135***	0.7807***	0.8642***	0.7574***	0.8852***
Leg length, cm	0.8098***	0.7308***	0.8095***	0.6115***	0.7703***	0.8437***	0.7454***	0.7946***
Thoracic depth, cm	0.7486***	0.6312***	0.7073***	0.6450***	0.7166***	0.7382***	0.6790***	0.7267***
Leg circumference, cm	0.8508***	0.7814***	0.7415***	0.6463***	0.8155***	0.8365***	0.7844***	0.8212***
Thoracic perimeter, cm	0.9218***	0.8846***	0.8528***	0.7277***	0.8911***	0.8911***	0.7683***	0.9307***
Carcass compactness index, kg / cm	0.8183***	0.8620***	0.7171***	0.6068***	0.8378***	0.7965***	0.6600***	0.8208***
Leg compactness index, kg / cm	0.2176^ns^	0.1404^ns^	0.0952^ns^	0.1767^ns^	0.1090^ns^	0.1119^ns^	0.0791^ns^	0.2393^ns^
LEAc, cm^2^	0.4390**	0.05914^ns^	0.0836^ns^	0.0793^ns^	0.1749^ns^	0.1952^ns^	- 0.00214^ns^	0.3565*

^ns^P > 0.05;

*P < 0.05;

**P < 0.01;

***P < 0.001.

LEAc: Loin eye area obtained in the carcass after cross sectioning of the *Longissimus lumborum*

Low coefficients of correlation were found between morphometric measurements and physical and chemical compositions of carcasses when expressed as percentages (Table 8). Therefore, they were not used in the prediction models.

**Table 8:** Correlation coefficients between carcass measurements and corrected proportions of physical and chemical compositions in cold carcasses of Santa Inês sheep.

Carcass measurements	Physical composition, %				Chemical composition, %			
	Muscle	Adipose	Bone	Other tissues	Fat	Protein	Ash	Water
**Weights**								
Hot carcass weight, kg	- 0.1602^ns^	0.7012***	- 0.6223***	- 0.097^ns^	0.7168***	0.0156^ns^	- 0.6986***	- 0.5390**
Cold carcass weight, kg	- 0.1753^ns^	0.7122***	- 0.6260***	- 0.0867^ns^	0.7224***	0.0189^ns^	- 0.6950***	- 0.5469**
**Carcass measurements**								
Carcass external length, cm	- 0.1026^ns^	0.5997***	- 0.5700***	- 0.0490^ns^	0.6640***	0.0726^ns^	- 0.5326**	- 0.5575***
Carcass internal length, cm	0.1216^ns^	- 0.0690^ns^	- 0.0211^ns^	0.0057^ns^	0.0546^ns^	0.0059^ns^	- 0.0333^ns^	- 0.0482^ns^
Croup width, cm	- 0.0097^ns^	0.4632**	- 0.4965**	- 0.0336^ns^	0.4185**	- 0.0130^ns^	- 0.5580***	- 0.2690^ns^
Thoracic width, cm	- 0.1733^ns^	0.4728**	- 0.4068**	0.0594^ns^	0.4460**	- 0.1292^ns^	- 0.5721***	- 0.2263^ns^
Croup perimeter, cm	- 0.1032^ns^	0.5307**	- 0.5170**	0.0288^ns^	0.5173**	0.0851^ns^	- 0.6142***	- 0.4065**
Leg length, cm	- 0.1818^ns^	0.5414**	- 0.4273**	- 0.0988^ns^	0.5533***	0.1695^ns^	- 0.5344**	- 0.5063**
Thoracic depth, cm	- 0.0417^ns^	0.4520**	- 0.4924**	0.0754^ns^	0.5734***	0.0954^ns^	- 0.4860**	- 0.4942***
Leg circumference, cm	- 0.0137^ns^	0.6771***	- 0.7327***	- 0.0276^ns^	0.7225***	0.1458^ns^	- 0.5134**	- 0.6591***
Thoracic circumference, cm	- 0.1855^ns^	0.6996***	- 0.6148***	- 0.0515^ns^	0.6820***	- 0.0193^ns^	- 0.7078***	- 0.4839**
Carcass compactness index, kg / cm	- 0.2158^ns^	0.7405***	- 0.6216***	- 0.0990^ns^	0.6962***	0.0070^ns^	- 0.6620***	- 0.5224**
Leg compactness index kg / cm	0.1761^ns^	0.0502^ns^	- 0.2110^ns^	0.0525^ns^	- 0.0142^ns^	- 0.1822^ns^	- 0.1694^ns^	0.1542^ns^
LEAc, cm^2^	0.3424^ns^	- 0.1921^ns^	- 0.2520^ns^	- 0.0626^ns^	- 0.0999^ns^	- 0.0746^ns^	- 0.2755^ns^	0.2068^ns^

^ns^P > 0.05;

**P < 0.01;

***P < 0.001.

LEAc: Loin eye area obtained in the carcass after cross sectioning of the *Longissimus lumborum*.

The parameters HCW (Table 9) and CCW (Table 10) were predictors of physical and chemical compositions of carcasses in the model. They presented high coefficients of determination, which varied in hot carcasses between 0.9769 and 0.7255 (Table 9) and in cold carcasses between 0.9813 and 0.7354 for water and ash levels (Table 10), respectively.

**Table 9:** Prediction equations for estimating the physical and chemical components in hot carcasses of Santa Inês sheep.

Dependent variable Y	Steps	Independent variable X	P	R^2^	RSD	Equations
Muscle, kg	1	HCW	< .0001	0.9759	0.0912	Y = 0.7256 + 0.5635H CW
	2	CWc	< .0001	0.9784	0.084	Y = − 1.50099 + 0.52724 HCW + 0.08957 CRW
Adipose, kg	1	HCW	< .0001	0.8512	0.0763	Y = − 0.80481 + 0.19354 HCW
	2	CCI	< .0001	0.8859	0.06	Y = − 0.8987 + 0.13791 HCW + 3.6072 CCI
	3	CP	< .0001	0.8975	0.0553	Y = 0.78654 + 0.16981 HCW—0.03663 CP + 4.15233 CCI
Bone, kg	1	HCW	< .0001	0.8611	0.053	Y = 0.48688 + 0.16794 HCW
	2	LL	< .0001	0.8702	0.0508	Y = − 0.86893 + 0.14390 HCW + 0.04221 LL
	3	ICL	< .0001	0.8786	0.0488	Y = − 1.46394 + 0.13564 HCW + 0.00845I CL + 0.04833LL
	4	TW	< .0001	0.8873	0.0465	Y = − 2.36418 + 0.10566 HCW + 0.0091 ICL + 0.04531 TW + 0.05602 LL
Fat, kg	1	HCW	< .0001	0.8996	0.0469	Y = − 0.63374 + 0.18991 HCW
	2	CCI	< .0001	0.9111	0.0426	Y = − 0.68536 + 0.15932 HCW + 1.98348 CCI
	3	CWc	< .0001	0.9233	0.0377	Y = 0.55104 + 0.18274 HCW—0.07089 CWc + 2.32591 CCI
	4	ICL	< .0001	0.9312	0.0347	Y = -2.40267–0.01013 HCW+ 0.05755 ICL -0.07266CWc + 12.77113 CCI
	5	HCW_removed_	< .0001	0.9312	0.0338	Y = − 2.24900 + 0.05463 ICL—0.07286 CWc + 12.23191 CCI
	6	CP	< .0001	0.939	0.0308	Y = − 1.51729 + 0.06054 ICL—0.05052 CWc—0.03106 CP + 13.64969 CCI
Protein, kg	1	HCW	< .0001	0.9137	0.0437	Y = − 0.0855 + 0.19929 HCW
	2	LL	< .0001	0.927	0.038	Y = − 1.97327 + 0.16582 HCW + 0.05877 LL
	3	LC	< .0001	0.9321	0.0362	Y = − 2.65792 + 0.14043 HCW + 0.06232 LL + 0.02356 LC
Ash, kg	1	HCW	< .0001	0.7255	0.0034	Y = 0.14317 + 0.02763 HCW
	2	ECL	< .0001	0.7497	0.0032	Y = − 0.22896 + 0.01901 HCW + 0.00894 ECL
Water, kg	1	HCW	< .0001	0.9769	0.0825	Y = 0.33232 + 0.5475 HCW
	2	TW	< .0001	0.982	0.066	Y = − 1.09583 + 0.48961 HCW + 0.10394 TW
	3	CP	< .0001	0.9842	0.0594	Y = -2.82860 + 0.45177 HCW + 0.08987 TW + 0.04151 CP

Steps: stepwise system steps; P: probability; R^2^: coefficient of determination; RSD: residual standard deviation; HCW: hot casting weight; CWc: croup width measured in the carcass; CCI: carcass compactness index; CP: croup perimeter; LL: leg length; TW: Thoracic width; ICL: internal carcass length; LC: leg circumference; ECL: external carcass length.

**Table 10:** Prediction equations for estimating the physical and chemical components in cold carcasses of Santa Inês sheep.

Dependent variable Y	Steps	Independent variable X	P	R^2^	RSD	Equations
Muscle, kg	1	CCW	< .0001	0.9768	0.0878	Y = 0.23671 + 0.5829 CCW
	2	CWc	< .0001	0.9784	0.0839	Y = − 1.05211 + 0.55208 CCW + 0.07306 CWc
Adipose, kg	1	CCW	< .0001	0.8615	0.0710	Y = -0.76543 + 0.20132CCW
	2	CCI	< .0001	0.8855	0.0602	Y = − 0.84424 + 0.1498 CCW + 3.15758 CCI
	3	CP	< .0001	0.9021	0.0529	Y = 1.25009 + 0.19505 CCW– 0.04536 CP + 3.60268 CCI
Bone,kg	1	CCW	< .0001	0.8684	0.0502	Y = 0.52583 + 0.17438 CCW
	2	CCI	< .0001	0.8818	0.0463	Y = 0.57661 + 0.20758 CCW—2.0347 CCI
	3	LC	< .0001	0.8929	0.0430	Y = 1.33005 + 0.24342 CCW– 0.03058 LC– 2.29853 CCI
Fat, kg	1	CCW	< .0001	0.9055	0.0441	Y = − 0.5868 + 0.19699 CCW
	2	CP	< .0001	0.9200	0.0383	Y = 1.26622 + 0.24322 CCW—0.03992 CP
	3	CCI	< .0001	0.9293	0.0348	Y = 1.4711 + 0.2185 CCW—0.04536 CP + 1.90068 CCI
	4	TD	< .0001	0.9351	0.0328	Y = 0.27971 + 0.18234 CCW—0.04774 CP + 0.06388 TD + 2.95849 CCI
	5	TW	< .0001	0.9388	0.0318	Y = 0.75520 + 0.20428 CCW– 0.03358 TW– 0.04375 CP + 0.05445 TD + 2.71389 CCI
Protein, kg	1	CCW	< .0001	0.9204	0.0403	Y = − 0.03754 + 0.20681 CCW
	2	LL	< .0001	0.9299	0.0364	Y = − 1.67321 + 0.17655 CCW + 0.05081 LL
Ash, kg	1	CCW	< .0001	0.7354	0.0033	Y = 0.14846 + 0.02876 CCW
	2	ECL	< .0001	0.7554	0.0031	Y = − 0.19537 + 0.02053 CCW + 0.00822 ECL
Water, kg	1	CCW	< .0001	0.9813	0.0669	Y = 0.47659 + 0.56735 CCW
	2	TW	< .0001	0.9856	0.0530	Y = − 0.8538 + 0.51208 CCW + 0.09579 TW

Steps: stepwise system steps; P: probability; R^2^: coefficient of determination; RSD: residual standard deviation; CCW: cold carcass weight; CWc: croup width measured in the carcass; CCI: carcass compactness index; CP: croup perimeter; LC: leg circumference; TD: thorax depth; TW: thorax width; LL: leg length; ECL: external carcass length.

In addition to HCW, CWc was added to the muscle tissue prediction model. For adipose tissue, CCI and CP were added. Regarding bone tissue estimation, the variables that were included in the model were LL, ICL, and TW, providing an increase in R^2^ from 0.8611 to 0.8873 (Table 9).

For the chemical composition, in addition to HCW, LL and LC were added to estimate the amount of protein in carcasses, ECL for ash, and TW and CP for water. To estimate the amount of fat, the HCW was removed in the fifth step, and the final model included CCI, CWc, ICL and CP, in which the R^2^ increased from 0.8996 to 0.9390 (Table 9).

Using CCW as an independent variable to replace HCW, we observed that, for the estimation of muscle, adipose tissues, and ash, the same independent variables were kept in the final model. However, for bone tissue, the CCW entered the model together with CCI and LC; for protein, CCW and LL were added. For water, CCW and TW were added. For fat estimation, CCW, CP, CCI, TD and TW were the independent variables of the prediction model (Table 10).

Taking into account the correlation results (Table 7), LEAc was placed together with the morphometric measurements in the model to assess its contribution in predicting the amount of muscle and water in the carcass. This measure entered the muscle tissue prediction model in the third step of the stepwise procedure for the HCW (Mt, kg = − 1.49247 + 0.42981 HCW + 0.03753 LEAc + 0.134141 CWc, R^2^ = 0.8902) and for the CCW (Mt, kg = − 1.111025 + 0.44174 CCW + 0.05653 LEAc + 0.11850 CWc, R^2^ = 0.8954).

## Discussion

The variable SBW is susceptible to external influences [3]. However, it is a performance and fattening indicator [17], and therefore, as the slaughter weight was not fixed, SBW showed a high variation (Table 1). In this sense, a diversified database is desirable for a better accuracy [18].

The variation observed in BL, TP_l_ and WH may be due to the different slaughter weights of the animals. The variation observed in BL may be due to the rate of the structures that make up the axial skeleton, since the neck and ribs present a growth curve equal to that of the carcass, whereas the loin has a late growth [19]. Therefore, they followed the variability between animals.

The leg cut has an isogonic growth in relation to the carcass [19], which may have contributed to the variability in CH, as occurred with body weight. However, the animals were homogeneous in terms of body shape, which is related to the low variability in the BCI (Table 1).

The strong correlation of SBW with the physical and chemical components of the carcass (Table 4) led to a greater influence of this variable in estimating the chemical and physical composition of carcasses, except for the estimate of the amount of Ash (Table 6). The SBW explained 89.47% of the variations regarding the amount of muscle, 81.40% of bone, 70.70% of adipose tissue, 81.49% of fat, 79.42% of protein, and 91.88% of water (Table 6). The coefficients of determination obtained to estimate the physical and chemical components of the carcass by ultrasound were lower than those obtained when carcass measurements were used for these estimates (Tables 9 and 10). However, they should not be disregarded, since biometric measurements can serve as a tool in the selection of animals for meat production.

The SBW is a simple, fast, and widely used measurement to assess animal performance [20] and an important biometric measure. It serves as a premise for the formation of batches in a production system [21], in addition to marketing purposes, since it is one of the main indicators of the CCW, acting both as a criterion for selection by producers and for sale in refrigerators [22].

This variable has a high correlation with biometric measurements that indicate meat production capacity [3]. However, the use of animal weight alone may overestimate or underestimate the actual composition of tissues in carcasses [9]. On the other hand, when associated with other biometric measurements, it can ensure better responses regarding the growth and development of the final product.

In predicting the weight of the muscle in carcasses, in addition to the SBW, two measurements were added to the model (BCI and ChW). The inclusion of these measures provided an increase in the coefficient of determination of 2.22%, reaching an accuracy of 91.46% (Table 6). The SBW, BCI and ChW strongly correlated with muscle tissue (Table 4), suggesting their use in assessing muscle mass.

The model for estimating the weight of adipose tissue was less accurate than that for muscle and bone tissues. In addition to the SBW, the ChW was included in the model and increased the coefficient of determination from 70.70 to 73.26% (Table 6).

The use of ChW to estimate adipose and muscle tissues is justified because the deposition of these tissues affects this measurement. However, when the BCI was added to the model, the respective regression coefficient was negative. Once the growth of the BL is stabilized, as bone growth stops and body weight continues to increase [3], and considering that the BCI is calculated by the relation between SBW and BL, the negative BCI in the model contributed to an improved accuracy in predicting muscle tissue.

The inclusion of WH in the second step of the stepwise procedure to estimate the amount of bone tissue provided an increase in the coefficient of determination from 81.40 to 84.82%. Of all physical and chemical components, bone tissue was the only one that correlated strongly with WH (Table 4). This is corroborated by the reports of Hammond [23] and Perez et al. [24], who stated that the cranial region of the animal’s body grows earlier than the caudal region.

Souza et al. [3] reported that limbs are the most promising regions when it is intended to estimate the amount of bone tissue in the carcass. Agamy et al. [25] observed a behavior similar to that obtained in the present study when they studied Ossimi sheep, but with an accuracy of 0.54.

It is worth mentioning that, by comparing the SBW regression coefficient to estimate muscle tissue (0.24782) with that to estimate adipose tissue (0.08461), and by comparing these with that to estimate bone tissue (0.08954) (Step 1 of Table 6), it is possible to infer that the deposition of muscle tissue was faster than that of bone tissue, and these tissues also deposited faster than adipose tissue. This may be because the animals were growing and because the PCA is a measurement that serves to estimate the composition of the body. However, its use in isolation is not recommended [9], since growth models may be responsible for different routes of tissue deposition [26], although the increase in weight gain is characterized by the deposition of bone, muscle, and adipose tissues, whose growth impulses vary in different parts of the body depending on age, race, production system, and diet [3, 9].

Among the chemical components of the carcass, only ash did not have an SBW as a good estimator, in addition to being the least accurate model (Table 6). To estimate it, the measurements that best fitted the model were CH and ChW, which explained 54.88% of the variation of this component in carcasses. Costa and Silva et al. [27], in a work of data compilation, reported an R^2^ of 0.40 to estimate ash in the carcass of beef cattle. Thus, the use of the model obtained in the present work can be recommended.

The CH was the measurement with the highest correlation with the amount of ash (Table 4). It is worth mentioning that the leg is the main representative of the CH measurement; this meat cut has a greater amount of bone in the carcass and of mineral constituents.

The model to estimate water was the most accurate one; 93.52% of its variation can be explained when SBW, ChW, and BCI are included. Water most concentrates in muscle tissue [28], which, in turn, can be found in at a greater proportion in the carcass (Table 2). In addition, these components had a stronger correlation (0.9761), as Table 3 shows, which resulted in the same predictive variables for muscle tissue and the amount of water.

Agamy et al. [25], using sheep of Egyptian breeds, also reported that biometric measurements did not fit the multivariate model to estimate the amount of fat, considering only the use of SBW as an independent variable (Table 6).

Fat is present in all tissues. Its largest proportion is in the adipose tissue, whose deposition rate in the carcass was lower than that of bone tissue. In addition, this is the most variable component in the animal’s body. The dynamics of its storage is overly complex [29] and has been modified with animal selection over time, further increasing the difficulties of its prediction [30, 31].

To estimate the amount of protein, the BCI improved the model’s accuracy by 2.47%. The negative regression coefficient of BCI in this model contributed similar to that of muscle tissue. This is plausible since these components are highly correlated (Table 3).

The lower coefficient of determination of the model using LEAu or LEAc, in comparison to those obtained without the referred measurement (Table 6 and Tables 9 and 10, respectively), was due to the smaller amount of data obtained in this experiment for this estimate (n = 33) because of the loss of some images, which provided less variability and resulted in a reduction in accuracy [18]. However, it is clear how much these measures can contribute to improving muscle tissue prediction because of the high determination coefficient obtained in the models. Additionally, Williams [32], in a literature review, reported that these LEA measurements, when taken between the 12th and 13th ribs, are strongly positively related to the distribution of muscles in the carcass. The models for prediction of the physical and chemical components of carcasses using morphometric measurements and the HCW (Table 9) or CCW (Table 10) showed a similar accuracy (R^2^) between the final models. In both options (Tables 9 and 10), the HCW or CCW entered the first step of the prediction equations.

For the estimation of muscle and fat tissues, the independent variables included in the model were the same, only differentiating HCW or CCW. The CWc was the measurement included in the model for predicting muscle tissue (Tables 9 and 10); its contribution to the accuracy of the models was lower than 0.3%. However, this measurement correlated strongly with muscle tissue (r = 0.8094). This corroborates Pinheiro and Jorge [33], who reported that CWc is an indicator of the proportion of muscles in the cut leg. Therefore, it is important in the evaluation of muscularity.

The CCI and CP were included in the model for estimating adipose tissue. They justified 89.75% of the variations when they entered together with the HCW (Table 9) and 90.21% when they entered together with the CCW (Table 10). The results obtained in the present study on the improvement of the determination coefficient when CP was included are consistent with those of Teixeira et al. [34], who reported that the base of the tail in sheep is a good predictor of body fat. These measurements contributed 5.44 and 4.71%, respectively, to the increase in R^2^.

The differences in the coefficients of determination were due to the morphometric measurements and because the estimates of the amount of fat were conducted in the carcass after cooling. However, they were small, which justifies the use of the HCW in situations where there is no way to subject them to a cold environment. Since HCW and CCW are positively and highly significantly intercorrelated (P < 0.001) [35, 36], it allows their use in the estimation of the chemical and physical components in carcasses.

To estimate bone tissue using the HCW, three independent variables were added to the multivariate model (LL, ICL and TW), improving accuracy by 3.04%, which resulted in an R^2^ of 88.73%. When the CCW was used, two measures were added (CCI and LC), improving the accuracy from 86.84 to 89.29%, that is, by 2.82%. The regression coefficient of the BCIc and LC for bone tissue estimation was negative (Table 10). It can be considered coherent, given that the CCI is more related to the deposition of muscle and fat tissue [37], which are also responsible for the increase in carcass weight.

For the chemical composition, only the amount of ash kept the same independent variables in the model that used the HCW and CCW (Tables 9 and 10). The inclusion of ECL contributed with approximately 3% in accuracy, with a final R^2^ of 74.97% with HCW and 75.54% with CCW.

Regarding the protein estimate in carcasses, the LL entered the two models. However, when the HCW was used, LC was added, which denotes that this variable can better predict the protein composition of carcasses since it is causally linked to muscle tissue, which is the tissue with the highest concentration of this component.

The chemical composition of the muscle greatly reflects the variation in tissue composition of the carcass. According to Gomide et al. [38], there is a definite parallel between the growth behavior of the chemical components of meat and the physically separated carcass tissues (muscles, bones, and fat), as already discussed regarding the relation between muscle tissues, protein, and water.

The addition of two measurements to the model with the HCW and one with the CCW provided little contribution in predicting the amount of water in carcasses. The increase in accuracy was 0.65 and 0.44%, respectively.

To estimate the amount of fat, five measurements were suggested to the model. However, only three would be necessary, that is, CCW, CP, and CCI would be the measurements that would most contribute to the improvement of the model (R^2^ = 0.9293). On the other hand, when there is only HCW, the stepwise procedure suggests the removal of this variable in the fifth step, maintaining CCI, CWc and ICL as independent variables of the fat quantity prediction model. Thus, the R^2^ is higher (0.9312). The use of HCW or CCW is not necessary, since the multivariate model with CCI, CWc, and ICL was the most accurate one.

In general, in certain circumstances, the inclusion of a variable did not represent an increase in accuracy. This would be a plausible justification for reducing the work to obtain it, especially if it was obtained from a different reference point [39]. However, according to Hopkins et al. [40], if additional precision is needed to estimate the physical or chemical composition of carcasses, a combination of measurements would justify the extra work and time.

## Conclusions

The biometric measurements of growing Santa Inês sheep can be used together with the slaughter body weight to estimate the physical and chemical compositions of carcasses, with emphasis on body compactness index, chest width, wither height, and croup height.

The morphometric measurements can be used together with carcass weight to estimate the physical and chemical compositions of carcasses, with emphasis on croup width, carcass compactness index, croup perimeter, external and internal carcass lengths, thoracic width, and leg length and perimeter.

The hot carcass weight can be used to predict the physical and chemical composition of carcasses, especially when it is not possible to obtain cold carcass weight, without compromising the accuracy of the prediction model.

## Supporting information

S1 Table Data that originated Table 1. (DOCX)

S2 Table Data that originated Table 2. (DOCX)

S1 Data (DOCX)
