# Supplementary material for: Prediction of carcass composition through measurements in vivo and measurements of the carcass of growing Santa Inês sheep
Source: PLoS One. 2021 Mar 5;16(3):e0247950. doi: 10.1371/journal.pone.0247950 (PMC7935253; doi:10.1371/journal.pone.0247950)
Supplement: S1 Table — (DOCX) [file pone.0247950.s001.docx]

**S1 Table 1. Data that originated Table 1.**

| Animal | Slaughter body weight | Croup height | Withers height | Body length | Chest width | Croup width | Thoracic perimeter | Body compactness index | Loin eye area obtained by ultrasound |
| --- | --- | --- | --- | --- | --- | --- | --- | --- | --- |
| 93 | 19.46 | 59 | 63 | 58 | 16 | 14 | 64 | 0.341 | - |
| 94 | 18.29 | 57 | 61 | 55 | 18 | 17 | 67 | 0.344 | - |
| 49 | 25.66 | 66 | 70 | 72 | 21 | 20 | 69 | 0.357 | - |
| 90 | 21.90 | 61 | 67 | 62 | 21.3 | 21 | 66 | 0.340 | - |
| 188 | 35.46 | 66 | 67 | 71 | 22 | 21.5 | 75 | 0.489 | - |
| 197 | 32.82 | 66 | 70 | 70 | 21.5 | 19 | 75 | 0.464 | - |
| 91 | 29.64 | 63 | 66 | 65 | 20.5 | 19.5 | 72 | 0.417 | 12.769 |
| 47 | 31.80 | 65 | 66 | 72 | 19.5 | 16.5 | 74 | 0.424 | 12.060 |
| 46 | 37.10 | 70 | 71 | 74 | 22 | 22.5 | 76 | 0.486 | 14.740 |
| 194 | 36.98 | 65 | 65 | 71 | 23.5 | 23.5 | 75 | 0.514 | 10.402 |
| 43 | 34.50 | 65 | 66 | 71 | 22 | 23 | 71 | 0.477 | 11.265 |
| 48 | 37.50 | 68 | 69 | 75 | 25.5 | 25 | 78 | 0.500 | 15.181 |
| 96 | 36.52 | 65 | 67 | 70 | 23.3 | 24 | 75 | 0.496 | 15.358 |
| 45 | 33.34 | 68 | 68 | 73 | 20.5 | 18 | 76 | 0.442 | 10.784 |
| 44 | 38.76 | 70 | 71 | 75 | 21 | 20.5 | 77 | 0.491 | 13.336 |
| 50 | 34.22 | 69 | 65 | 69 | 20 | 21 | 71 | 0.459 | 11.656 |
| 99 | 31.70 | 68 | 66 | 71 | 21 | 20 | 74 | 0.446 | 16.194 |
| 41 | 35.34 | 73 | 69 | 71 | 22.5 | 21 | 78 | 0.496 | 12.728 |
| 98 | 39.99 | 69 | 71 | 75 | 25.5 | 25 | 76 | 0.520 | 15.163 |
| 182 | 40.14 | 71 | 69 | 72 | 23.5 | 24.5 | 76 | 0.524 | 17.084 |
| 193 | 38.00 | 73 | 70 | 74 | 25 | 24 | 73 | 0.504 | 13.237 |
| 198 | 35.64 | 68 | 67 | 71 | 23 | 28 | 76 | 0.496 | 11.529 |
| 100 | 35.14 | 70 | 70 | 71 | 21 | 21 | 75 | 0.472 | 12.010 |
| 190 | 33.54 | 67 | 68 | 69 | 22 | 19 | 75 | 0.486 | 10.418 |
| 92 | 35.36 | 68 | 67 | 74 | 20.5 | 22.5 | 72 | 0.461 | 10.003 |
| 195 | 38.70 | 73 | 72 | 76 | 23.5 | 21.5 | 75 | 0.509 | 12.937 |
| 183 | 38.20 | 70 | 67 | 70 | 22 | 21.5 | 77 | 0.536 | 12.855 |
| 192 | 35.90 | 67.5 | 68 | 70 | 24 | 23.5 | 76 | 0.497 | 15.417 |
| 181 | 39.50 | 70 | 71 | 70 | 24 | 21 | 76 | 0.546 | 14.836 |
| 199 | 33.70 | 67 | 71 | 73 | 22.5 | 21 | 74 | 0.445 | 12.596 |
| 186 | 34.72 | 66 | 68 | 67 | 23 | 21 | 76 | 0.516 | 12.882 |
| 189 | 32.18 | 67 | 65 | 69 | 21.5 | 20.5 | 71.5 | 0.466 | 13.927 |
| 42 | 36.38 | 67 | 71 | 74 | 21.5 | 20.5 | 75 | 0.477 | 12.496 |
| 185 | 35.44 | 70 | 70 | 76 | 22 | 20.5 | 76 | 0.453 | 12.564 |
| 184 | 39.02 | 70.5 | 69 | 71 | 24 | 21.5 | 77 | 0.528 | 12.642 |
| 51 | 36.84 | 69 | 67 | 70 | 23.5 | 23 | 76 | 0.514 | 11.883 |
| 89 | 40.50 | 69 | 70 | 79 | 24 | 22.5 | 81 | 0.505 | 15.458 |
| 87 | 34.40 | 66 | 65 | 63 | 21.3 | 21 | 74 | 0.524 | 9.203 |
| 187 | 35.70 | 66 | 69 | 77 | 22.5 | 20 | 75 | 0.453 | 11.697 |
