# Supplementary material for: Prediction of carcass composition through measurements in vivo and measurements of the carcass of growing Santa Inês sheep
Source: PLoS One. 2021 Mar 5;16(3):e0247950. doi: 10.1371/journal.pone.0247950 (PMC7935253; doi:10.1371/journal.pone.0247950)
Supplement: S2 Table — (DOCX) [file pone.0247950.s002.docx]

**S2 Table 2. Data that originated Table 2 part 1.**

| Animal | Hot carcass weight. kg | Cold carcass weight. kg | External carcass length. cm | Internal carcass length. cm | Croup width. cm | Thoracic width. cm | Croup perimeter. cm |
| --- | --- | --- | --- | --- | --- | --- | --- |
| 88 | 7.320 | 6.700 | 48.0 | 51.0 | 19.5 | 19.5 | 57.0 |
| 95 | 9.180 | 8.480 | 49.0 | 54.0 | 23.0 | 20.0 | 61.0 |
| 97 | 6.140 | 5.540 | 45.0 | 50.0 | 20.5 | 18.0 | 50.5 |
| 93 | 9.580 | 9.538 | 49.0 | 53.0 | 22.5 | 18.5 | 56.0 |
| 94 | 9.044 | 8.264 | 52.0 | 56.0 | 22.0 | 18.0 | 56.5 |
| 49 | 12.764 | 12.474 | 56.0 | 46.0 | 22.0 | 20.0 | 60.5 |
| 90 | 10.908 | 10.188 | 55.0 | 60.0 | 21.5 | 18.5 | 56.5 |
| 188 | 17.678 | 16.618 | 58.0 | 61.0 | 24.5 | 23.5 | 64.5 |
| 197 | 16.406 | 15.606 | 55.0 | 45.0 | 24.0 | 21.5 | 64.5 |
| 91 | 15.526 | 14.586 | 56.0 | 62.0 | 23.5 | 21.0 | 62.0 |
| 47 | 15.740 | 15.028 | 55.0 | 49.0 | 21.5 | 20.5 | 64.0 |
| 46 | 18.220 | 17.322 | 61.0 | 52.0 | 25.0 | 22.5 | 66.0 |
| 194 | 17.740 | 17.034 | 56.0 | 50.0 | 26.0 | 25.5 | 67.0 |
| 43 | 16.180 | 15.438 | 61.0 | 64.0 | 25.0 | 23.5 | 63.0 |
| 48 | 19.295 | 17.780 | 56.0 | 65.0 | 24.0 | 25.0 | 63.0 |
| 96 | 17.940 | 17.068 | 59.0 | 49.0 | 25.0 | 22.0 | 66.5 |
| 45 | 15.506 | 14.676 | 60.0 | 50.0 | 23.0 | 23.0 | 62.5 |
| 44 | 18.910 | 18.170 | 62.0 | 51.0 | 27.0 | 25.0 | 66.0 |
| 50 | 16.244 | 15.524 | 58.0 | 51.0 | 24.0 | 21.0 | 66.0 |
| 99 | 16.378 | 14.940 | 57.0 | 63.0 | 23.5 | 21.0 | 61.0 |
| 41 | 17.310 | 16.510 | 57.0 | 63.0 | 24.0 | 22.0 | 69.5 |
| 98 | 18.966 | 18.266 | 59.0 | 55.0 | 25.0 | 26.5 | 69.0 |
| 182 | 19.206 | 18.446 | 58.0 | 53.0 | 27.0 | 25.0 | 70.0 |
| 193 | 19.262 | 18.382 | 63.0 | 68.0 | 25.0 | 24.5 | 66.0 |
| 198 | 17.000 | 16.240 | 59.0 | 66.0 | 23.0 | 23.0 | 64.5 |
| 100 | 16.926 | 15.946 | 59.0 | 61.0 | 22.0 | 22.0 | 61.0 |
| 190 | 15.426 | 14.786 | 57.0 | 61.0 | 23.5 | 22.5 | 63.0 |
| 92 | 16.926 | 16.166 | 59.0 | 58.0 | 24.0 | 24.0 | 65.5 |
| 195 | 19.382 | 17.780 | 58.0 | 65.0 | 26.0 | 24.5 | 66.0 |
| 183 | 18.044 | 17.284 | 60.0 | 48.0 | 25.5 | 23.5 | 65.0 |
| 192 | 17.312 | 16.612 | 58.0 | 52.0 | 27.0 | 25.5 | 69.0 |
| 181 | 19.160 | 18.460 | 58.0 | 65.0 | 25.0 | 25.0 | 69.0 |
| 199 | 16.420 | 15.580 | 60.0 | 49.0 | 24.0 | 21.0 | 64.5 |
| 186 | 16.586 | 15.946 | 57.0 | 48.0 | 23.0 | 24.0 | 67.0 |
| 189 | 16.404 | 15.060 | 58.0 | 64.0 | 24.0 | 24.0 | 61.5 |
| 42 | 17.258 | 16.618 | 59.0 | 62.0 | 24.0 | 24.0 | 65.0 |
| 185 | 16.462 | 15.562 | 59.0 | 67.0 | 24.5 | 22.5 | 65.0 |
| 184 | 18.678 | 17.938 | 59.0 | 53.0 | 26.0 | 25.0 | 68.0 |
| 51 | 16.710 | 15.830 | 55.0 | 52.0 | 25.5 | 26.5 | 65.0 |
| 89 | 19.282 | 18.402 | 62.0 | 68.0 | 26.0 | 23.5 | 71.0 |
| 87 | 15.234 | 14.454 | 57.0 | 47.0 | 23.0 | 22.0 | 61.0 |
| 187 | 17.706 | 16.906 | 58.0 | 53.0 | 26.0 | 23.5 | 69.0 |

**S2 Table 2. Data that originated Table 2 part 2.**

| Animal | Leg length. cm | Thoracic depth.cm | Leg circumference. cm | Thoracic perimeter. cm | Carcass compactness index. kg / cm | Leg compactness index. kg / cm | Loin eye area obtained in the carcass |
| --- | --- | --- | --- | --- | --- | --- | --- |
| 88 | 36.0 | 23.0 | 28.5 | 59.0 | 13.137 | 54.167 | - |
| 95 | 39.0 | 22.5 | 33.0 | 60.0 | 15.704 | 58.974 | - |
| 97 | 36.0 | 21.0 | 24.0 | 56.5 | 11.080 | 56.944 | - |
| 93 | 39.0 | 23.0 | 39.0 | 62.5 | 17.996 | 57.692 | - |
| 94 | 35.0 | 23.0 | 39.0 | 57.0 | 14.757 | 62.857 | - |
| 49 | 41.0 | 23.0 | 35.0 | 63.5 | 27.117 | 53.659 | - |
| 90 | 37.0 | 24.0 | 36.0 | 63.5 | 16.980 | 58.108 | - |
| 188 | 45.5 | 25.0 | 41.0 | 71.0 | 27.243 | 53.846 | - |
| 197 | 43.0 | 25.0 | 40.0 | 70.5 | 34.680 | 55.814 | - |
| 91 | 40.0 | 26.0 | 41.0 | 69.0 | 23.526 | 58.750 | 15.559 |
| 47 | 43.0 | 24.0 | 41.0 | 66.5 | 30.669 | 50.000 | 14.917 |
| 46 | 41.0 | 25.0 | 39.0 | 72.0 | 33.312 | 60.976 | 15.896 |
| 194 | 40.0 | 24.0 | 42.0 | 74.0 | 34.068 | 65.000 | 11.636 |
| 43 | 41.5 | 26.0 | 44.0 | 68.0 | 24.122 | 60.241 | 13.397 |
| 48 | 41.0 | 26.5 | 42.0 | 68.0 | 27.354 | 58.537 | 15.610 |
| 96 | 42.0 | 25.0 | 41.0 | 71.5 | 34.833 | 59.524 | 15.073 |
| 45 | 41.0 | 25.0 | 41.0 | 69.0 | 29.352 | 56.098 | 10.129 |
| 44 | 45.0 | 26.0 | 42.0 | 75.0 | 35.627 | 60.000 | 13.586 |
| 50 | 42.0 | 26.0 | 40.5 | 68.5 | 30.439 | 57.143 | 11.536 |
| 99 | 41.0 | 26.0 | 39.5 | 68.5 | 23.714 | 57.317 | 17.857 |
| 41 | 43.0 | 26.0 | 41.5 | 70.5 | 26.206 | 55.814 | 13.067 |
| 98 | 42.0 | 26.0 | 42.5 | 73.0 | 33.211 | 59.524 | 14.969 |
| 182 | 43.0 | 26.0 | 46.0 | 74.5 | 34.804 | 62.791 | 15.728 |
| 193 | 44.0 | 26.0 | 44.0 | 74.0 | 27.032 | 56.818 | 11.262 |
| 198 | 39.0 | 28.0 | 40.0 | 72.0 | 24.606 | 58.974 | 11.359 |
| 100 | 42.0 | 25.0 | 43.0 | 70.0 | 26.141 | 52.381 | 13.695 |
| 190 | 40.0 | 25.0 | 39.0 | 70.5 | 24.239 | 58.750 | 11.075 |
| 92 | 41.0 | 25.0 | 41.0 | 70.5 | 27.872 | 58.537 | 11.115 |
| 195 | 44.0 | 26.0 | 42.0 | 70.0 | 27.354 | 59.091 | 13.306 |
| 183 | 41.0 | 25.0 | 43.0 | 70.0 | 36.008 | 62.195 | 12.362 |
| 192 | 41.0 | 25.0 | 41.0 | 73.0 | 31.946 | 65.854 | 15.433 |
| 181 | 44.0 | 27.0 | 41.0 | 71.0 | 28.400 | 56.818 | 16.015 |
| 199 | 42.0 | 23.0 | 38.5 | 67.5 | 31.796 | 57.143 | 13.617 |
| 186 | 43.0 | 26.0 | 41.0 | 71.5 | 33.221 | 53.488 | 10.664 |
| 189 | 39.0 | 25.0 | 42.0 | 67.0 | 23.531 | 61.538 | 15.185 |
| 42 | 42.0 | 27.0 | 44.0 | 71.0 | 26.803 | 57.143 | 12.405 |
| 185 | 42.0 | 27.0 | 39.0 | 70.0 | 23.227 | 58.333 | 11.632 |
| 184 | 44.0 | 25.0 | 42.0 | 72.5 | 33.845 | 59.091 | 13.084 |
| 51 | 41.0 | 24.0 | 41.0 | 72.0 | 30.442 | 62.195 | 13.645 |
| 89 | 44.0 | 28.0 | 44.0 | 73.0 | 27.062 | 59.091 | 19.162 |
| 87 | 40.0 | 26.0 | 39.0 | 70.5 | 30.753 | 57.500 | 10.654 |
| 187 | 42.0 | 25.0 | 41.0 | 73.0 | 31.898 | 61.905 | 12.923 |

**S2 Table 2. Data that originated Table 2 part 3.**

| Animal | Muscle tissue. kg | Adipose tissue. kg | Bone tissue. kg | Other tissue. kg | Muscle tissue. % | Adipose tissue. % | Bone tissue. % | Other tissues. % |
| --- | --- | --- | --- | --- | --- | --- | --- | --- |
| 88 | 4.098 | 0.524 | 1.692 | 0.386 | 61.157 | 7.819 | 25.259 | 5.765 |
| 95 | 5.182 | 0.708 | 2.238 | 0.352 | 61.114 | 8.343 | 26.387 | 4.156 |
| 97 | 3.137 | 0.349 | 1.853 | 0.201 | 56.620 | 6.296 | 33.453 | 3.631 |
| 93 | 5.712 | 1.647 | 1.816 | 0.363 | 59.891 | 17.272 | 19.035 | 3.803 |
| 94 | 5.087 | 0.833 | 1.878 | 0.466 | 61.558 | 10.075 | 22.725 | 5.642 |
| 49 | 7.621 | 1.413 | 2.920 | 0.521 | 61.096 | 11.326 | 23.405 | 4.173 |
| 90 | 6.628 | 0.905 | 2.264 | 0.391 | 65.057 | 8.881 | 22.223 | 3.839 |
| 188 | 10.070 | 2.529 | 3.432 | 0.587 | 60.600 | 15.217 | 20.651 | 3.532 |
| 197 | 9.043 | 2.801 | 3.112 | 0.650 | 57.945 | 17.948 | 19.940 | 4.167 |
| 91 | 9.375 | 2.199 | 2.642 | 0.370 | 64.273 | 15.076 | 18.115 | 2.535 |
| 47 | 9.101 | 2.344 | 3.091 | 0.492 | 60.557 | 15.595 | 20.571 | 3.276 |
| 46 | 9.771 | 3.091 | 3.635 | 0.825 | 56.411 | 17.842 | 20.985 | 4.763 |
| 194 | 9.801 | 2.730 | 3.675 | 0.829 | 57.537 | 16.027 | 21.572 | 4.865 |
| 43 | 9.144 | 1.938 | 3.496 | 0.860 | 59.231 | 12.550 | 22.647 | 5.572 |
| 48 | 10.669 | 3.061 | 3.409 | 0.641 | 60.006 | 17.214 | 19.176 | 3.604 |
| 96 | 10.190 | 2.717 | 3.356 | 0.805 | 59.701 | 15.920 | 19.664 | 4.714 |
| 45 | 8.348 | 2.593 | 3.067 | 0.668 | 56.880 | 17.667 | 20.899 | 4.554 |
| 44 | 11.059 | 2.770 | 3.490 | 0.852 | 60.866 | 15.242 | 19.205 | 4.687 |
| 50 | 9.747 | 2.150 | 3.012 | 0.614 | 62.787 | 13.851 | 19.404 | 3.958 |
| 99 | 9.212 | 2.173 | 2.964 | 0.591 | 61.660 | 14.545 | 19.842 | 3.953 |
| 41 | 9.634 | 2.482 | 3.704 | 0.690 | 58.352 | 15.033 | 22.433 | 4.181 |
| 98 | 11.132 | 2.571 | 3.846 | 0.717 | 60.943 | 14.075 | 21.054 | 3.928 |
| 182 | 11.290 | 2.877 | 3.379 | 0.901 | 61.204 | 15.595 | 18.317 | 4.883 |
| 193 | 10.998 | 2.770 | 3.976 | 0.638 | 59.831 | 15.071 | 21.629 | 3.468 |
| 198 | 9.396 | 2.578 | 3.473 | 0.793 | 57.860 | 15.874 | 21.386 | 4.880 |
| 100 | 9.013 | 2.828 | 3.428 | 0.677 | 56.523 | 17.735 | 21.496 | 4.246 |
| 190 | 9.007 | 2.159 | 3.157 | 0.463 | 60.918 | 14.598 | 21.354 | 3.130 |
| 92 | 9.337 | 2.790 | 3.288 | 0.751 | 57.758 | 17.257 | 20.338 | 4.648 |
| 195 | 10.719 | 2.337 | 3.979 | 0.745 | 60.287 | 13.147 | 22.379 | 4.187 |
| 183 | 10.318 | 3.060 | 3.496 | 0.410 | 59.695 | 17.707 | 20.228 | 2.369 |
| 192 | 10.451 | 2.491 | 3.145 | 0.524 | 62.915 | 14.996 | 18.931 | 3.157 |
| 181 | 10.651 | 2.935 | 3.879 | 0.995 | 57.698 | 15.901 | 21.013 | 5.388 |
| 199 | 9.333 | 2.674 | 3.032 | 0.541 | 59.904 | 17.165 | 19.459 | 3.471 |
| 186 | 9.480 | 2.389 | 3.410 | 0.667 | 59.453 | 14.979 | 21.384 | 4.184 |
| 189 | 9.515 | 2.156 | 2.947 | 0.443 | 63.179 | 14.315 | 19.568 | 2.938 |
| 42 | 9.625 | 2.265 | 3.830 | 0.898 | 57.920 | 13.629 | 23.046 | 5.405 |
| 185 | 9.414 | 2.234 | 3.274 | 0.639 | 60.494 | 14.358 | 21.040 | 4.108 |
| 184 | 10.361 | 2.884 | 3.965 | 0.728 | 57.760 | 16.077 | 22.107 | 4.056 |
| 51 | 9.569 | 2.270 | 3.178 | 0.813 | 60.448 | 14.339 | 20.077 | 5.136 |
| 89 | 11.124 | 2.549 | 3.950 | 0.779 | 60.452 | 13.850 | 21.463 | 4.236 |
| 87 | 8.450 | 2.676 | 2.726 | 0.602 | 58.464 | 18.511 | 18.861 | 4.163 |
| 187 | 10.113 | 2.879 | 3.266 | 0.647 | 59.821 | 17.030 | 19.320 | 3.829 |

**S2 Table 2. Data that originated Table 2 part 4.**

| Animal | Fat. kg | Protein. kg | Ash. kg | Water. kg | Fat. % | Protein. % | Ash. % | Water. % |
| --- | --- | --- | --- | --- | --- | --- | --- | --- |
| 88 | 0.696 | 1.433 | 0.311 | 4.260 | 10.394 | 21.389 | 4.634 | 63.583 |
| 95 | 0.876 | 1.606 | 0.355 | 5.643 | 10.330 | 18.942 | 4.181 | 66.548 |
| 97 | 0.379 | 1.092 | 0.289 | 3.781 | 6.837 | 19.702 | 5.219 | 68.241 |
| 93 | 1.366 | 2.060 | 0.476 | 5.636 | 14.323 | 21.601 | 4.987 | 59.090 |
| 94 | 1.079 | 1.941 | 0.452 | 4.792 | 13.062 | 23.483 | 5.475 | 57.981 |
| 49 | 1.838 | 2.351 | 0.546 | 7.739 | 14.732 | 18.848 | 4.380 | 62.040 |
| 90 | 1.384 | 1.798 | 0.485 | 6.521 | 13.582 | 17.651 | 4.760 | 64.007 |
| 188 | 2.816 | 3.386 | 0.520 | 9.896 | 16.947 | 20.376 | 3.128 | 59.549 |
| 197 | 2.878 | 3.357 | 0.550 | 8.822 | 18.442 | 21.508 | 3.522 | 56.528 |
| 91 | 2.355 | 2.948 | 0.467 | 8.816 | 16.148 | 20.208 | 3.200 | 60.443 |
| 47 | 2.498 | 3.295 | 0.594 | 8.641 | 16.622 | 21.924 | 3.955 | 57.499 |
| 46 | 2.911 | 3.505 | 0.608 | 10.298 | 16.808 | 20.236 | 3.507 | 59.449 |
| 194 | 2.931 | 3.349 | 0.571 | 10.182 | 17.209 | 19.663 | 3.354 | 59.774 |
| 43 | 2.333 | 3.285 | 0.597 | 9.222 | 15.115 | 21.281 | 3.869 | 59.735 |
| 48 | 3.025 | 3.584 | 0.619 | 10.552 | 17.016 | 20.156 | 3.481 | 59.346 |
| 96 | 2.974 | 3.115 | 0.598 | 10.381 | 17.423 | 18.251 | 3.503 | 60.824 |
| 45 | 2.251 | 3.140 | 0.536 | 8.749 | 15.337 | 21.393 | 3.653 | 59.617 |
| 44 | 3.019 | 3.581 | 0.640 | 10.930 | 16.614 | 19.709 | 3.520 | 60.156 |
| 50 | 2.261 | 3.404 | 0.606 | 9.253 | 14.564 | 21.928 | 3.904 | 59.605 |
| 99 | 2.128 | 3.227 | 0.559 | 9.026 | 14.240 | 21.602 | 3.740 | 60.418 |
| 41 | 2.645 | 3.280 | 0.573 | 10.012 | 16.021 | 19.869 | 3.468 | 60.641 |
| 98 | 2.591 | 3.714 | 0.680 | 11.280 | 14.186 | 20.335 | 3.725 | 61.755 |
| 182 | 2.892 | 3.857 | 0.629 | 11.068 | 15.680 | 20.909 | 3.411 | 60.001 |
| 193 | 2.703 | 4.259 | 0.818 | 10.602 | 14.706 | 23.169 | 4.448 | 57.678 |
| 198 | 2.667 | 3.177 | 0.594 | 9.802 | 16.425 | 19.560 | 3.656 | 60.359 |
| 100 | 2.888 | 3.210 | 0.640 | 9.207 | 18.113 | 20.133 | 4.014 | 57.740 |
| 190 | 2.403 | 2.891 | 0.537 | 8.955 | 16.251 | 19.550 | 3.633 | 60.566 |
| 92 | 2.542 | 3.248 | 0.668 | 9.708 | 15.725 | 20.092 | 4.134 | 60.050 |
| 195 | 2.742 | 3.779 | 0.773 | 10.486 | 15.421 | 21.257 | 4.347 | 58.975 |
| 183 | 3.077 | 3.368 | 0.629 | 10.210 | 17.805 | 19.484 | 3.637 | 59.074 |
| 192 | 2.287 | 3.433 | 0.636 | 10.256 | 13.766 | 20.663 | 3.831 | 61.740 |
| 181 | 3.134 | 3.550 | 0.661 | 11.115 | 16.978 | 19.230 | 3.581 | 60.211 |
| 199 | 2.572 | 3.384 | 0.605 | 9.018 | 16.510 | 21.721 | 3.885 | 57.884 |
| 186 | 2.427 | 3.459 | 0.751 | 9.309 | 15.222 | 21.689 | 4.709 | 58.380 |
| 189 | 2.375 | 2.549 | 0.572 | 9.565 | 15.768 | 16.926 | 3.796 | 63.510 |
| 42 | 2.759 | 3.342 | 0.681 | 9.836 | 16.601 | 20.111 | 4.096 | 59.191 |
| 185 | 2.492 | 3.279 | 0.657 | 9.133 | 16.015 | 21.073 | 4.225 | 58.687 |
| 184 | 2.848 | 3.762 | 0.686 | 10.642 | 15.875 | 20.973 | 3.827 | 59.326 |
| 51 | 2.537 | 2.869 | 0.582 | 9.843 | 16.025 | 18.121 | 3.675 | 62.179 |
| 89 | 3.065 | 4.035 | 0.731 | 10.571 | 16.657 | 21.927 | 3.972 | 57.444 |
| 87 | 2.960 | 2.919 | 0.500 | 8.076 | 20.478 | 20.194 | 3.457 | 55.872 |
| 187 | 2.464 | 3.585 | 0.611 | 10.245 | 14.573 | 21.208 | 3.617 | 60.603 |
