# Supplementary material for: Prediction of carcass composition through measurements in vivo and measurements of the carcass of growing Santa Inês sheep
Source: PLoS One. 2021 Mar 5;16(3):e0247950. doi: 10.1371/journal.pone.0247950 (PMC7935253; doi:10.1371/journal.pone.0247950)
Supplement: S1 Data — (DOCX) [file pone.0247950.s003.docx]

**S3 Table 3. Data that originated Table 3:** For Table 3 we used data from animals: 88, 95, 97, 93, 94, 49, 90, 188, 197, 91, 47, 46, 194, 43, 48, 96, 45, 44, 50, 99, 41, 98, 182, 193, 198, 100, 190, 92, 195, 183, 192, 181, 199, 186, 189, 42, 185, 184, 51, 89, 87, and 187.

**S4 Tables 4, 5, and 6. Data that originated Tables 4, 5, and 6:** For Tables 4, 5 and 6 we used data from animals: 93, 94, 49, 90, 188, 197, 91, 47, 46, 194, 43, 48, 96, 45, 44, 50, 99, 41, 98, 182, 193, 198, 100, 190, 92, 195, 183, 192, 181, 199, 186, 189, 42, 185, 184, 51, 89, 87, and 187, except the loin eye area obtained by ultrasound in the *Longissimus lumborum* muscle, where the data used were from animals 91, 47, 46, 194, 43, 48, 96, 45, 44, 50, 99, 41, 98, 182, 193, 198, 100, 190, 92, 195, 183, 192, 181, 199, 186, 189, 42, 185, 184, 51, 89, 87, and 187.

**S5 Tables 7, 8, 9, and 10. Data that originated Tables 7, 8, 9, and 10:** For Table 7, 8, 9, and 10 we used data from animals: 88, 95, 97, 93, 94, 49, 90, 188, 197, 91, 47, 46, 194, 43, 48, 96, 45, 44, 50, 99, 41, 98, 182, 193, 198, 100, 190, 92, 195, 183, 192, 181, 199, 186, 189, 42, 185, 184, 51, 89, 87, and 187, except the Loin eye area obtained in the carcass after cross sectioning of the *Longissimus lumborum* muscle, where the data used were from animals 91, 47, 46, 194, 43, 48, 96, 45, 44, 50, 99, 41, 98, 182, 193, 198, 100, 190, 92, 195, 183, 192, 181, 199, 186, 189, 42, 185, 184, 51, 89, 87, and 187.
